# The effect of systemic factors on retinal blood flow in patients with carotid stenosis: an optical coherence tomography angiography study

**DOI:** 10.1007/s11357-021-00492-1

**Published:** 2021-11-27

**Authors:** Lilla István, Cecilia Czakó, Fruzsina Benyó, Ágnes Élő, Zsuzsa Mihály, Péter Sótonyi, Andrea Varga, Zoltán Zsolt Nagy, Illés Kovács

**Affiliations:** 1 Department of Ophthalmology, Semmelweis University, 39 Mária Street, 1085 Budapest, Hungary; 2 Department of Vascular & Endovascular Surgery, Semmelweis University, Budapest, Hungary; 3 Department of Ophthalmology, Weill Cornell Medical College, New York, USA; 4 Department of Clinical Ophthalmology, Faculty of Health Sciences, Semmelweis University, Budapest, Hungary

**Keywords:** Retinal biomarkers, Carotid artery stenosis, Retinal imaging, OCT angiography

## Abstract

Carotid artery stenosis (CAS) is among the leading causes of mortality and permanent disabilities in the Western world. CAS is a consequence of systemic atherosclerotic disease affecting the majority of the aging population. Optical coherence tomography angiography (OCTA) is a novel imaging technique for visualizing retinal blood flow. It is a noninvasive, fast method for qualitative and quantitative assessment of the microcirculation. Cerebral and retinal circulation share similar anatomy, physiology, and embryology; thus, retinal microvasculature provides a unique opportunity to study the pathogenesis of cerebral small vessel disease in vivo. In this study, we aimed to analyze the effect of systemic risk factors on retinal blood flow in the eyes of patients with significant carotid artery stenosis using OCT angiography. A total of 112 eyes of 56 patients with significant carotid stenosis were included in the study. We found that several systemic factors, such as decreased estimated glomerular filtration rate (eGFR), hypertension, and carotid occlusion have a significant negative effect on retinal blood flow, while statin use and carotid surgery substantially improve ocular microcirculation. Neither diabetes, clopidogrel or acetylsalicylic acid use, BMI, serum lipid level, nor thrombocyte count showed a significant effect on ocular blood flow. Our results demonstrate that a systematic connection does exist between certain systemic risk factors and retinal blood flow in this patient population. OCTA could help in the assessment of cerebral circulation of patients with CAS due to its ability to detect subtle changes in retinal microcirculation that is considered to represent changes in intracranial blood flow.

## Introduction

Carotid artery stenosis (CAS) is among the most important health concerns as it frequently leads to transient ischemic attacks (TIA) or ischemic stroke—these being one of the leading causes of mortality and permanent disabilities [1, 2]. The principal risk factors for carotid artery disease are being aged over 50, hyperlipidemia, smoking, coronary disease, peripheral artery disease, stroke, or TIA in the patient’s medical history, or the occurrence of a cardiovascular event in family members younger than 60 years [3]. Treatment can be through conservative or surgical therapy. Conservative therapy is recommended in asymptomatic cases when the extent of the stenosis is lower than 70% or when the risk of an intervention is too high compared to its potential benefits. If surgery is required, endarterectomy is the preferred treatment option due to the lower perioperative stroke rate [4, 5].

The preferred diagnostic method for CAS in current practice is Duplex ultrasound, which provides morphological information and allows one to quantify the extent of the stenosis. Although it can be a reliable examination method if performed by a trained examiner, several human factors can result in an increase of variability and error during ultrasound examinations [6, 7]. CT or MR angiography are more objective methods that are mostly used in reconstruction planning.

Through thromboembolic and hemodynamic mechanisms, CAS can also lead to severe ophthalmic complications, which are often a predictor for the occurrence of cerebrovascular accidents. Transient monocular visual loss (amaurosis fugax) is caused by a temporary decrease in blood flow and usually lasts from seconds to minutes, though in rare cases, can persist for hours [8]. The most common cause is embolization from the ipsilateral carotid artery resulting in the ischemia of the retina, the choroid, or the optic nerve; that said, hypotension or vasospasm can also lead to hypoperfusion [9]. Embolization can also lead to sudden, painless, but permanent blindness due to a retinal stroke [10].

The examination of ophthalmic complications of CAS is conventionally possible by slit-lamp examination and funduscopy. Optical coherence tomography angiography is a new method for visualizing and analyzing the retinal and choroidal vasculature without the use of intravenous dye [11]. With the use of OCT angiography, subtle changes in retinal blood flow can be detected, with high accuracy. Owing to the short acquisition time, and since it is noninvasive, OCTA is beneficial to patient comfort and can be repeated at any time during follow-up visits. Numerous studies have described the high accuracy and reproducibility of OCTA parameters in normal subjects [12–19], as well as in patients with diabetes [20], glaucoma [21], ischemic optic neuropathy, [22] and retinal vascular diseases [23, 24]. However, it is known, that image quality significantly affects both the measurement error [25–29] alongside the OCTA parameters [30] suggesting the need to use a correction factor for longitudinal analysis.

In a recent study, Lahme et al. described reduced retinal flow density in CAS patients compared to healthy controls, and a significant improvement in the capillary network was also noted after surgery using OCTA [31]. Furthermore, Lee et al. found a significant increase in the vessel density of the macular deep vessel complex on both eyes after surgical procedure for CAS [32]. Although these studies reported an improvement in retinal blood flow after carotid endarterectomy, none of them took into consideration the effect of systemic risk factors or image quality on retinal blood flow measurements.

The purpose of this study was to analyze the effect of systemic risk factors on retinal blood flow in eyes of patients with significant carotid artery stenosis using OCT angiography before and after carotid surgery.

## Methods

In this prospective clinical study, a total of 56 patients with significant carotid artery stenosis were enrolled, all of them prepared to undergo carotid endarterectomy at the Department of Vascular & Endovascular Surgery at Semmelweis University. The study followed the tenets of the Declaration of Helsinki’s applicable national and local requirements and was approved prospectively by the Ethical Review Board for Human Research of the National Drug Agency. All participants gave their written informed consent.

Inclusion criteria for the study were twofold: significant carotid artery stenosis (≥ 70%) and planned endarterectomy. Exclusion criteria were associated with ocular disease (such as age-related macular degeneration, glaucoma, or vitreomacular disease), previous intraocular anti-VEGF injection, and the presence of clinically significant media opacities.

The severity of carotid artery stenosis was assessed by computed tomography angiography (CTA) as part of the clinical routine. All carotid patients underwent preoperative, unenhanced cranial 256-slice scanner CT. CTA of the carotid arteries was performed from the level of the aortic arch to the vertex using bolus tracking (Brilliance iCT 256, Philips Healthcare, Best, The Netherlands). The following imaging parameters were used for data acquisition: 120 kV, 50–160 mAs/slice, slice thickness 0.67 mm, Philips® IMR reconstruction, an intravenous contrast agent (Iomeron400), 50 ml at a flow rate of 5 ml/sec.

### Definitions of patient characteristics

Several separate patient characteristics were measured—specifically carotid artery stenosis, hypertension, diabetes, patients’ treatment, chronic kidney disease, eGFR—as follows: unequivocal diagnosis of significant carotid artery stenosis and the indication for reconstruction was determined through CT angiography based on the NASCET criteria [33]. Hypertension was defined according to the 2020 American Heart Association (AHA) guidelines [34], while diabetes was defined in line with the 2019 ESC guideline [35], and the patients’ treatment was described by their general practitioner before the procedures. Chronic kidney disease (CKD) was defined as the preoperative estimated glomerular filtration rate (eGFR; ml/min/1.73m^2^) < 60 according to the Kidney Disease: Improving Global Outcomes (KDIGO)[36]. The eGFR was calculated with reference to an average person with a body surface of 1.73 m^2^ and was not corrected for gender or body mass index (BMI). The best medical therapy was administered for all patients after the result of the CT angiography (Aspirin 100 mg once or Clopidogrel 75 mg once with a statin) according to the European Society for Vascular Surgery (ESVS) guidelines [37].

### Surgical procedure

Surgical treatment was performed at Semmelweis University Department of Vascular & Endovascular Surgery. All patients underwent carotid artery reconstruction under general anesthesia. The incision made was longitudinal, parallel to the medial border of the sternocleidomastoid muscle, the carotid sheath being entered, and the medial border of the jugular vein dissected. Most of the patients underwent eversion endarterectomy, which was performed with the complete transection of the bifurcation; followed by everting the adventitia and mobilizing it upward while gentle caudad traction was applied to the plaque. After the endarterectomy, the divided bifurcation was reunited with a simple end-to-end anastomosis. The patients who were selected for shunting with Pruitt–Inawera shunt, underwent a conventional endarterectomy, which consists of a vertical arteriotomy from CCA (common carotid artery) to ICA (internal carotid artery) and closure using bovine patch angioplasty. The shunting was indicated preoperatively based on the Circle of Willis (CoW) status. Near-infrared spectroscopy (NIRS) was used for cerebral monitoring.

### Optical coherence tomography angiography

**Fig. 1:**
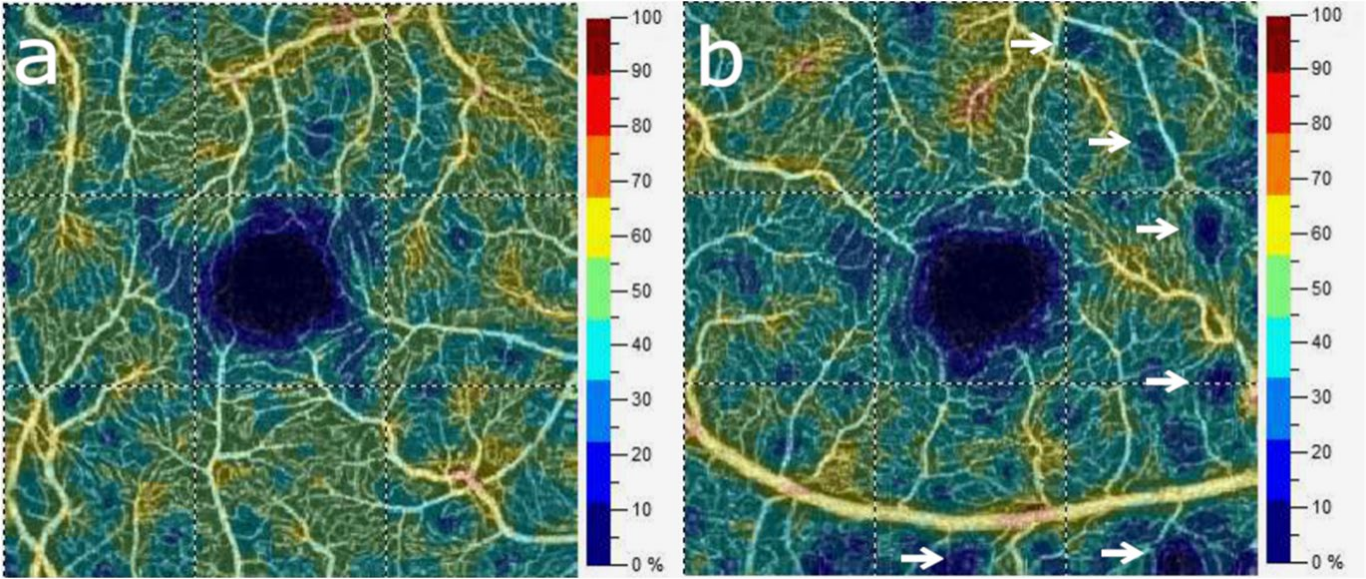
Color-coded images of macular vessel density (VD) from both eyes of a patient with left unilateral carotid artery stenosis show preserved retinal blood flow (VD: 44.7%) on the contralateral right eye (**a**) and areas of non-perfusion (arrows) and decreased overall perfusion (VD:41.9%) in the ipsilateral left eye (**b**)

Each study subject underwent three sessions of imaging, during which three OCTA images of the macular area and three images of the optic nerve head were obtained consecutively. The first session was scheduled during the perioperative period, the other two during the first postoperative week and one month following surgery. Ophthalmologic visits consisted of testing visual acuity, with slit lamp and fundus examinations followed by optical coherence tomography angiography imaging. Optical coherence tomography angiography imaging was performed under the same conditions by a trained examiner. OCTA imaging was performed using an AngioVue device with an SSADA (split-spectrum amplitude-decorrelation angiography) software algorithm (RTVue XR Avanti with AngioVue, Optovue Inc, Fremont, CA, USA). The device obtains 70,000 A-scans per second in approximately 3.0 s. Imaging of the macula required a 3 × 3-mm scan as the current version of AngioAnalytics software acquires scans with the highest resolution in the central 3-mm diameter. Images with movement artifacts (such as vessel doubling, white line artifacts, vessel discontinuities, or noise), projection artifacts, and segmentation errors were excluded. Scan quality (SQ) was required to be above SQ 5, images with an SQ of 5 or below were also excluded. An example of macular vessel density in both eyes of a patient with significant left carotid stenosis is demonstrated in Fig. 1.

### Statistical analysis

Statistical analysis was performed with SPSS software (version 23.0, IBM, Armonk, NY, USA). The effect of systemic parameters, as well as image quality on OCTA parameters, was assessed with multivariable regression analysis using generalized estimating equation (GEE) models. In addition to treating repeated measurements (intrasession and between visits), this test enables adjustments to be made for within-subject correlation of parameters (right vs. left eye) by taking into account between-eye correlations. Moreover, the inclusion of scan quality and risk factors as covariates into GEE models permits one to simultaneously control for their effect on the dependent variables. Covariates evaluated as potential confounders included age, degree of stenosis in %, aspirin, clopidogrel, and statin use, BMI, eGFR, and serum cholesterol, low-density lipoprotein (LDL), high-density lipoprotein (HDL), triglyceride level, in addition to the presence of hypertension and diabetes. The effect of systemic predictors on OCTA parameters was analyzed first in bivariable models (adjusting for scan quality for each predictor) and statistically significant predictors entered in multivariable models. The construction of the multivariable regression model began with variables that showed the best fit to data in bivariable modeling—assessed using the value of the corrected quasi-likelihood under independence model criterion (QICC); where lower QICC values indicate a better fit to data. Following this, variables were added and the change in the QICC value was tested. Variables were kept in the model if they were associated with a *p* value less than 0.01 and the overall fit of the model improved, as indicated by a decrease in the QICC value compared with the value in a model that did not include the variable.

## Results

A total of 112 eyes of 56 patients (26 male and 30 female, mean age: 69.89 ± 7.07 years) were included in the study, the baseline characteristics of the study group are summarized in Table 1.

**Table 1:** Baseline characteristics of the study group

	Mean ± SD	Min–max
Age (years)	69.89 ± 7.07	53–84
Gender (F/M)	22/34	-
Carotid stenosis (%)	79.80 ± 8.96	70–99
Hpertension (Y/N)	52/4	-
Diabetes (Y/N)	21/35	-
Acetyl salicylic acid use (Y/N)	33/23	-
Clopidogrel use (Y/N)	21/35	-
Statin use (Y/N)	33/23	-
BMI	28.48 ± 5.64	18.31–42.24
ThCT (g/l)	244.02 ± 60.78	77.00–434.00
Creatinin (umol/l)	92.43 ± 40.31	46–257
eGFR (ml/min/1.73 m^2^)	69.82 ± 18.61	22–90
Cholesterol (mmol/l)	4.57 ± 1.49	2.20–9.70
HDL (mmol/l)	1.16 ± 0.26	0.61–2.08
LDL (mmol/l)	2.65 ± 1.07	0.81–6.30
Triglyceride (mmol/l)	1.98 ± 1.14	0.60–6.88

The values of SQ ranged from 6 to 10, the overall mean SQ was (7.45 ± 1.01), and there was no statistically significant difference between the SQ values of the ipsilateral and contralateral side (7.49 ± 0.99 vs. 7.41 ± 1.01; *p* = 0.12). Scan quality was found to be a significant predictor of superficial capillary vessel density (2.52%; 95%CI: 2.33–2.73%; *p* < 0.001); thus, scan quality values associated with each measurement were included as confounders in all statistical calculations.

On the eyes ipsilateral to the stenosis, morphological abnormalities—such as capillary dropout areas or deformation and enlargement of the foveal avascular zone (FAZ)—were visible on most of the angiograms. Figure 1 demonstrates mottled areas of capillary loss on the affected side of a patient with unilateral carotid artery stenosis. Although the AngioAnalytics software does not provide any quantitative information about the size of patchy capillary dropout, its summarized impact on macular capillary blood flow is represented in the overall vascular density value.

**Fig. 2:**
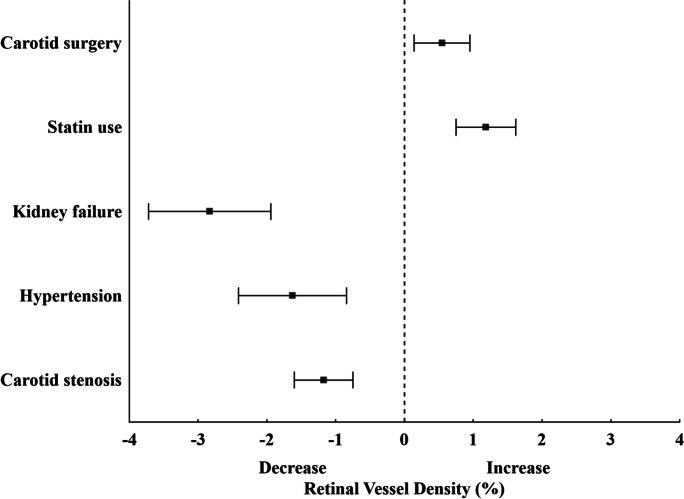
Forest plot demonstrates the effect estimates of systemic predictors and carotid surgery on retinal blood flow after adjustment for scan quality

Subsequently, we evaluated the effect of age, degree of stenosis in %, aspirin, clopidogrel and statin use, BMI, eGFR, serum cholesterol, low-density lipoprotein (LDL), high-density lipoprotein (HDL), triglyceride level, and the presence of hypertension and diabetes. Table 2 summarizes significant predictors of macular vessel density, both in bivariable models and in multivariable regression models. In bivariable regression models (adjusting for the effect of scan quality for each predictor separately), the presence of hypertension showed the greatest effect on vessel density (− 1.56%) followed by statin use (+ 0.95%), carotid surgery (+ 0.54%), higher eGFR (0.34% for 10 units), and carotid stenosis (− 0.11% for 10% stenosis). Age, aspirin and clopidogrel use, BMI, serum cholesterol, LDL, HDL, and triglyceride level or diabetes did not prove to be significant predictors of macular vessel density (*p* > 0.05). All significant predictors in bivariable models also remained significant predictors of vessel density in the final multivariable model (Table 2). Interestingly, statin use showed a marked positive influence on retinal blood flow, after controlling for the negative effect of hypertension, carotid occlusion, and lower eGFR in these patients both before and after carotid surgery (Table 2). At the first postoperative visit, we observed a significant increase in macular vessel density from baseline values in both the ipsi- and the contralateral eyes with no further improvement until the last measurement is taken 1 month after the intervention. There was no significant difference in the increase of vessel density between the two eyes of the same patient across the postoperative time points. The relative effect of significant predictors on retinal blood flow is summarized in Fig. 2.

**Table 2:** Significant predictors of macular vessel density in patients with CAS

	Superficial capillary layer					
	Bivariable analysis			Multivariable analysis		
	Beta	95% CI	*p*	Beta	95% CI	*p*
Scan quality (unit)	2.52	2.33–2.73	< 0.001	2.16	1.96–2.37	< 0.001
Carotid stenosis (10%)	− 0.11	− 0.05 to − 0.17	0.001	− 0.10	− 0.04 to − 0.16	0.001
Hypertension (Y/N)	− 1.56	− 0.77 to − 2.34	< 0.001	− 1.46	− 0.68 to − 2.23	< 0.001
eGFR (10 units)	0.34	0.23–0.45	< 0.001	0.35	0.24–0.46	< 0.001
Statin use (Y/N)	0.95	0.53–1.37	< 0.001	1.02	0.60–1.44	< 0.001
Surgery	0.54	0.12–0.96	0.01	0.55	0.14–0.95	0.01
Age (years)	− 0.12	− 0.09 to − 0.15	< 0.001	− 0.08	− 0.06 to − 0.12	< 0.001

Bivariable models included scan quality and one predictor. Multivariable models included all listed predictors

## Discussion

In the current study, we analyzed the qualitative and quantitative changes in the retinal circulation of patients with severe carotid artery stenosis. We found that several systemic factors, such as decreased eGFR, hypertension, and carotid occlusion have a significant negative effect on retinal blood flow, while statin use and carotid surgery substantially improve ocular blood flow. Confirming previous results, image quality had a statistically significant impact on vessel density and should be taken into consideration in analyzing retinal blood flow using OCT angiography. We found that carotid surgery resulted in a significant improvement in retinal blood flow both ipsi- and contralaterally, independently of systemic factors. It is interesting that in this patient group neither diabetes, clopidogrel or acetylsalicylic acid use, BMI, serum lipid level, nor thrombocyte count showed a significant effect on ocular blood flow. One explanation for this observation is that the strength of association between these risk factors and retinal blood flow decreases with simultaneous analysis of strong predictors, such as hypertension, statin use, or carotid surgery.

Hypertension affects the microcirculation by causing structural changes of the microvasculature through two mechanisms: remodeling of resistance vessels and causing capillary rarefaction [38]. Despite the underlying molecular mechanisms not being fully understood yet, studies suggest that prolonged vasoconstriction, reactive oxygen species (ROS), nitric oxide, matrix metalloproteinases (MMP), and tissue type transglutaminase (TG2) all play a role—leading to the remodeling of the arterioles through activation of cytoskeletal and extracellular matrix structures of the vessel wall [39]. Capillary rarefaction, with the loss of terminal arterioles and capillaries, is also highly associated with the presence of hypertension [40]. It is believed that an imbalance between increased vascular destruction and insufficient angiogenesis leads to its development [33, 41–45]. What is more, these changes have been observed in the cerebral circulation. The first study to report the changes in cerebral blood flow in patients with essential hypertension found higher limits in the cerebral blood flow autoregulation compared to a normotensive control group [46]. Since which, numerous other study groups have revealed alterations in the lumen diameter and the vessel number in the cerebral circulation [47–49]. Several studies reported microvascular changes of the retina associated with chronic hypertension. Greater intercapillary distance, reduced capillary area, and reduced retinal capillary flow were detected with the use of Doppler flowmetry [50, 51]. OCTA is also used in the examination of hypertensive patients. Decreased vessel density values were found in both the superficial and deep layers of the retinal capillary plexus; furthermore, the foveal avascular zone was found to have increased both in size and its perimeter compared to a normotensive control group [52, 53]. However, none of those studies controlled for the effect of scan quality on retinal blood flow measurements. It has to be emphasized that disregarding the relationship between OCTA scan quality and retinal vessel density could lead to erroneous conclusions being drawn from detected differences when comparing two study groups or when measuring fluctuations in retinal blood flow during consecutive measurements in these patients.

A correlation between lower eGFR values and impaired cerebral blood flow was previously reported in a population-based study [54]. Others have found an association between kidney function and subclinical markers of cerebral small vessel disease by evaluating MRI scans [55]. Previously, Zhouang et al. found decreased vessel density values in the superficial vascular complex correlated with decreased eGFR in diabetic patients using OCT angiography [56], but this is the first study that describes the significant association between eGFR and retinal blood flow in patients with CAS. The results of this study demonstrate that there is a strong correlation between generalized microvascular problems and renal function, although it is still a point of contention whether microcirculatory changes are a cause or the effect of decreased kidney function. Several studies have described a connection between coronary microvascular dysfunction and kidney disease [57–61]. Notably, Castro et al. found a correlation between impaired cerebral autoregulation and decreased kidney function in patients with acute ischemic stroke [62].

In the current study, we found a beneficial effect of statin use on retinal blood flow. HMG-CoA (3-hydroxy3-methylglutaryl coenzyme A) reductase inhibitors or statins are considered to be a first-line treatment in most cases of CAS as they have been proven to slow down, or may even partially reverse, atherosclerosis. They also appear to improve peripheral endothelial function and decrease carotid intima-media thickness [63]. In addition to lowering LDL-cholesterol levels, statins also have an anti-inflammatory effect, inhibit cell proliferation, promote apoptosis, and have an anticoagulant and antithrombotic effect [64]. Although there are still scant data concerning the effect of cholesterol-lowering therapy on peripheral microvasculature, some studies have confirmed that they are beneficial in regards to improving microcirculation [65, 66]. In animal models, statins were found to increase cerebral blood flow through nitric oxide (NO)-dependent and NO-independent mechanisms [67–69]. On the other hand, in human studies, the protective effect of statins was found to be related more to vasoreactivity than to improving cerebral blood flow [70–72]. What is more, a recent study found a connection between statin administration and the improvement of capillary rarefaction and microcirculation [73, 74]. As our results demonstrate, OCTA can be a great help in collecting further information with respect to the changes of microcirculation in connection with statin administration and in possibly verifying these previous findings.

There are several direct and indirect methods for assessing cerebral blood flow, though they all have their limitations. Of the direct methods, the most important are single-photon emission computed tomography (SPECT), positron emission tomography (PET), MRI with contrast agents, and arterial spin labeling (ASL) MRI [75]. The majority of these are based on measuring the amount of a tracer is delivered to brain tissue. As such, these are invasive examination methods which are difficult to implement and require complex, expensive equipment. ASL is based on the detection of magnetically labeled water molecules in arterial blood and is a promising new, noninvasive method for measuring intracranial blood flow. The most commonly used indirect methods are transcranial Doppler (TCD) ultrasound imaging, near-infrared spectroscopy (NIRS), and phase-contrast MRI. TCD is a noninvasive examination method during which the flow velocities in the major cerebral arteries are measured through the thinner areas of the skull using an ultrasound probe. The main limitations to TCD are that the accuracy of the measurements is highly operator-dependent and some patients are unsuitable due to inadequate acoustic windows [76]. NIRS is based on the absorption of near-infrared light and exploits the different optical properties of oxygenated and deoxygenated hemoglobin, thus the measurement depends on changes in both the blood flow and oxygen metabolism [76] Phase-contrasting MRI is an accurate and easily reproducible procedure, however, it is only suitable in the assessment of mean overall blood flow [77]. It is important to note that the indirect methods have the disadvantage of being unable to provide information on the flow of arterial blood to the capillary beds.

Being part of the central nervous system, the retina offers a unique and easy method to study cerebral small vessel diseases in vivo. Over the past few decades, a number of novel approaches in retinal imaging have been developed that may allow physicians and researchers to gain insights into the genesis and progression of cerebrovascular pathologies. Previously, apart from knowing the medical history, conventional examination methods helped the diagnostics of ocular complications of vascular diseases such as slit-lamp examination, funduscopy, and fluorescein angiography, which is an invasive technology for visualizing the retinal vasculature [78]. Optical coherence tomography (OCT) helps in visualizing the retinal structures by using low coherence interferometry [79]. High-resolution cross-sectional images can be obtained of the retina which allows in vivo visualization of the different retinal layers. OCT angiography is a fast and noninvasive procedure and is suitable for follow-up of conditions affecting the retinal blood flow. The imaging technique is based on motion contrast technology to detect retinal blood flow. It also enables quantitative measurement of the blood flow in the area of the macula and the optic nerve head, in addition to accurate visualization of microvascular abnormalities and capillary dropout areas in retinal vascular diseases. The software calculates a signal quality score for every scan, based on the signal intensity of the image acquired by the device using different ranges and recommended thresholds provided by the manufacturer. Numerous factors affect the scan quality including media opacities, blink artifacts, ocular saccades, and OCT operator skills. The Scan Quality index is a unitless parameter in the range of 0 to 10 (the larger the better) produced by the RTVue-XR AngioVue software with errors coming from eye motion, defocus, and signal-to-noise ratio incorporated in its formulation. Since image quality significantly affects OCTA parameters, this value has to be taken into consideration during the comparison of OCTA images for reliable results.

The use of OCT angiography—a new, noninvasive, and easily repeatable imaging technique—gives us an opportunity to detect early signs of retinal microvascular abnormalities, which could well mirror changes in the intracranial blood flow. Some previous studies have examined the changes in ophthalmological parameters in patients with CAS in which both morphological and functional changes were found compared to healthy control groups or between the two eyes of patients with unilateral CAS. Havelius et al. compared dark adaptation levels of patients with unilateral CAS to a healthy control group and found that dark adaptation levels were significantly lower in the patient group but found no difference between the two eyes [80]. In another study, functional changes were assessed together with blood flow alterations. Ocular pneumoplethysmography was performed in order to evaluate ophthalmic artery systolic pressure and retinal function was tested through multifocal electroretinography (mfERG) in patients with unilateral stenosis and no manifest ocular complication. They found significantly delayed and reduced electroretinographic responses in the ipsilateral eye. Furthermore, a correlation was observed between the mfERG results and the arterial blood pressure [81]. Sayin et al. evaluated spectral domain optical coherence tomography (SD-OCT) scans of patients with CAS and found a significant decrease in choroidal thickness compared to healthy controls. In the same study, the retinal nerve fiber layer (RNFL) thickness and macular and ganglion cell complex (GCC) thickness were evaluated, with no significant difference being found between the two study groups [82]. Wang et al. observed a correlation between the thinning of the RNFL layer and the presence of asymptomatic carotid artery stenosis by evaluating SD-OCT measurements of each patient [83]. Another study detected a thinning of the average RNFL thickness and macular thickness (MT) in the nine Early Treatment Diabetic Retinopathy Study (ETDRS) areas compared to a healthy control group [84]. In contrast, Heßler et al. assessed the different morphological and functional changes in the retina (such as RNFL thickness, total macular volume, optic nerve head volume, and visual acuity) and found no changes compared to a control group [85]. Other study groups examined the effect of carotid endarterectomy on ocular parameters. Yan et al. studied both subjective and objective visual functions—such as visual acuity, visually evoked potential, electroretinographic parameters, static, and kinetic visual field—and found a significant improvement in them [86]. Although they found no change in RNFL thickness, others could detect a significant decrease in peripapillary RNFL thickness after surgery [87]. Another study described a significant increase in choroidal thickness in patients with a 50–70% stenosis evaluating enhanced depth imaging optical coherence tomography (EDI-OCT) images [88].

In a recent study, Lahme et al. measured retinal flow density values in OCTA images and described a reduced flow density in CAS patients compared to healthy controls, and they did not find any difference between the ipsilateral and the contralateral eye. A significant improvement in the radial peripapillary capillary network (RPC) was also noted but no significant change was seen in superficial or deep layers of the macular area [31]. Lee et al. also performed OCTA examinations and evaluated the changes in retinal microcirculation before and after surgical procedure for CAS and found a significant increase in the vessel density of the macular deep vessel complex on both eyes. On the contralateral eye, the vessel density also improved in the superficial layer [32]. That said, in addition to having taken no notice of the effect of image quality on OCTA parameters, no previous study has evaluated the effect of systemic factors on retinal blood flow data from the two eyes of the same subject.

One of the limitations of this study is that the acquired data was obtained using a specific type of device restricting the generalizability of our results. However, the findings clearly show that a systematic connection does exist between certain systemic risk factors and retinal blood flow in this patient population. Further studies with larger cohorts of patients are recommended to validate these associations and to assess their relation to intracranial blood flow. Finally, whereas image quality-adjusted OCTA values are associated with improved comparability of scans, it needs to be confirmed whether the detection of changes in retinal microcirculation could help in the assessment of intracranial blood flow in patients with carotid occlusion. Nevertheless, any advancement that improves the ability to detect true changes in intracranial blood flow over time is valuable, and future studies could help to assess the role of OCTA metrics in the clinical setting. We are of the opinion that the implementation of OCTA imaging in the diagnostic armamentarium may well result in increased accuracy of the assessment of progression and treatment of these patients.
